# Clinical and Genetic Spectrum of Filippi Syndrome: A Systematic Review of Published Case Reports and Case Series

**DOI:** 10.1002/hsr2.72655

**Published:** 2026-07-04

**Authors:** Muhammad Anas Faheem, Laraib Ghanghro, Ayesha Khan, Maryam Khalid, Salwa Bashir, Dania Iqbal, Adan Ali, Hussain Haider Shah, Sameer Abdul Rauf, Humaira Kalam

**Affiliations:** ^1^ Department of Internal Medicine, Dow Medical College Dow University of Health Sciences Karachi Pakistan; ^2^ Dow University of Health Sciences Karachi Pakistan; ^3^ Department of Internal Medicine Liaquat University of Medical and Health Sciences (LUMHS), Jamshoro Pakistan; ^4^ Department of Internal Medicine, Allama Iqbal Medical College University of Health Sciences Lahore Pakistan; ^5^ Department of Internal Medicine, Ziauddin Medical College Ziauddin University Karachi Pakistan; ^6^ Liaquat National Medical Hospital Karachi Pakistan; ^7^ Voice of Doctors Research School Dhaka Bangladesh; ^8^ Shaheed Tajuddin Ahmad Medical College Gazipur Bangladesh

## Abstract

**Background and Aims:**

Filippi syndrome is a very rare autosomal recessive craniodigital disorder primarily caused by mutations in the gene CKAP2L, characterized by syndactyly, microcephaly, growth retardation, distinctive craniofacial features, and intellectual disability. Because of its rarity and phenotypical overlap and related craniodigital disorders, its full clinical and genetic spectrum remains poorly defined. This systematic review aimed to synthesize the published literature on the clinical presentation, genetic findings, and reported management of Filippi syndrome and Filippi‐like overlapping phenotypes.

**Methods:**

We performed a systematic review in accordance with PRISMA 2020, searching in PubMed, Embase, and Web of Science from inception to June 2025 for case reports and series describing Filippi syndrome, clinically diagnosed classical Filippi syndrome, and Filippi‐like overlapping phenotypes. Clinical, radiological, and genetic data were extracted descriptively.

**Results:**

Twenty‐two studies comprising 43 reported patients were included. The most frequent findings were characteristic craniofacial dysmorphism, digital syndactyly, microcephaly, short stature or growth retardation, and neurodevelopmental impairment. *CKAP2L* was the principal implicated gene in molecularly confirmed cases, while a smaller number of reports described alternative genetic findings in patients with Filippi‐like overlapping phenotypes. Additional manifestations included dental anomalies, skeletal abnormalities, neurologic involvement, cardiovascular defects, and genitourinary abnormalities. Reported management was mainly supportive and symptom‐directed, including surgical correction of syndactyly and rehabilitative therapies.

**Conclusion:**

Filippi syndrome has a broad and heterogenous clinical presentation centered on microcephaly, syndactyly, craniofacial dysmorphism, growth impairment, and developmental delay. Inclusion of Filippi‐like overlapping phenotypes highlights both the diagnostic complexity and the need to distinguish CKAP2L‐related disease from phenotypically similar entities. This review collates a broad overview of clinical and genetic observations, highlighting the necessity for a multidisciplinary approach to enhance management and outcome. Further studies will be needed to delineate genotype‐phenotype correlation and standardize treatment guidelines.

## 1 Introduction

Filippi syndrome is a very rare autosomal recessive disorder that causes syndactyly of the fingers and toes, microcephaly, slow growth, a unique craniofacial appearance, and intellectual disability. Most of the time, this is caused by changes in the *CKAP2L* gene [1, 2]. It was first described by G. Filippi in 1985 [3], with fewer than 50 cases reported globally, underscoring its rarity and clinical significance.

The existing literature is primarily comprised of isolated case reports and small case series dispersed across various journals that lack standardized documentation, complicating the ability to draw definitive conclusions. Moreover, there are no established diagnostic criteria or evidence‐based clinical management guidelines, which further complicates differential diagnosis due to the phenotypic overlap of Filippi syndrome with other craniodigital syndromes like Scott craniodigital syndrome and Chitayat syndrome [4, 5]. Additionally, the clinical heterogeneity, such as inconsistent reports of seizures, hearing loss, or urinary system abnormalities suggests a broader clinical spectrum [6, 7]. The paucity of detailed research into its natural history, genotype‐phenotype correlations, long‐term outcomes, and effective therapeutic strategy further underscores the need for comprehensive analyses.

In the context of rare genetic disorders such as Filippi syndrome, the individual case reports are the only available clinical evidence, functioning as building blocks to expand the knowledge base. Through the aggregation and evaluation of published case reports, consistent phenotypic features, recurrent patterns, and novel genetic mutations can now be recognized. Apart from assisting clinicians to confidently diagnose the syndrome, systematic reviews also guide geneticists and researchers to pursue targeted research or interventions. Furthermore, the analysis of case reports can reveal demographic patterns and also emphasize potential therapeutic targets, clinical interventions, and desirable outcomes.

The objective of this systematic review is to evaluate and synthesize the clinical characteristics described in the published case reports of Filippi syndrome to establish criteria for recognizing prevalent diagnostic approaches and phenotypic manifestations. It also offers to analyze the results in the context of the given management options available, which will be very helpful for doctors. Consolidating available information will create a more structured understanding of the syndrome. This will not only help us act quickly, but it will also show us areas that need more research.

## 2 Methods

### 2.1 Design and Registration

We conducted this systematic review in accordance with the PRISMA 2020 guidelines [8] and with a prior protocol registered on PROSPERO (International Prospective Register of Systematic Reviews) (CRD: 420251065218) [9], thereby ensuring transparency and prespecification of methods. The completed PRISMA 2020 checklist is provided in Supporting Material 1.

### 2.2 Search Strategy

We conducted an extensive and exhaustive search of case reports and series from inception till June 2025 from PubMed, Embase, and Web of Science. The search terms included “Filippi syndrome” OR “Filippi's syndrome” OR Filippi* NEAR/3 syndrome OR “microcephaly‐syndactyly syndrome” OR *CKAP2L* OR C5orf31 OR “cytoskeleton associated protein 2 like.” All English publications describing case reports and case series of confirmed Filippi Syndrome were included. Two independent reviewers comprehensively searched and filtered the eligible studies. We excluded all review articles, other similar presenting syndromes, and unconfirmed cases of Filippi Syndrome. The full electronic search strategy (including Boolean operators and MeSH/Emtree terms) is detailed in Supporting Material 2.

### 2.3 Inclusion Criteria

We added original case reports, case series, and editorials reporting cases that detailed human patients with Filippi syndrome or Filippi‐like phenotypes. A patient was eligible if: (i) biallelic pathogenic or likely‐pathogenic variants in *CKAP2L* were reported or (ii) the clinical presentation met the classical triad of microcephaly, digital syndactyly, and typical facies along with at least one other major feature (postnatal growth retardation, short stature, or dental anomalies) or (iii) the case was described by the original authors as Filippi‐like or as an overlapping craniodigital phenotype with substantial clinical resemblance to Filippi syndrome, even in the presence of an alternative genetic finding. Articles needed to report patient‐level clinical data on one or more of the outcomes of interest (growth parameters, neurologic findings, skeletal anomalies, cardiovascular or genitourinary abnormalities, radiologic data, or molecular results).

### 2.4 Study Selection and Data Extraction

All articles were imported into Rayyan and two reviewers independently screened the full text of each potentially relevant article and, for every study that met the eligibility criteria, extracted a predefined set of variables into a standardized spreadsheet. The variables captured comprised: the first author's last name, journal title, year of publication, study design, total number of Filippi‐syndrome patients, individual patient age and sex, detailed anthropometric measurements, evidence of intrauterine growth restriction (IUGR) and postnatal growth retardation, parental consanguinity and/or carrier‐mutation status, presence of affected siblings, inferred pattern of inheritance, results of any genetic analysis, cognitive or mental‐retardation level, developmental delay, seizures, dystonia or other neurologic signs, radiologic findings, speech or hearing status, digital syndactyly, microcephaly, short stature, dental anomalies, characteristic craniofacial features (eyes, nose, mouth), and any additional skeletal, cardiovascular, genitourinary or miscellaneous clinical features reported.

### 2.5 Assessment of Risk of Bias

Using the Joanna Briggs Institute (JBI) Checklist for Case reports and Case series, analysis by two independent authors determined the level of bias that existed among the studies included in the review. This checklist for case reports was divided into eight distinct domains, while that of case series consisted of nine domains. For each study, this tool was applied, and the presence of bias was categorized as yes, no, unclear, or not applicable, ultimately determining the level of bias risk as high, low, or with some concerns. Any inconsistencies that were discovered were discussed with a third author to find a solution.

### 2.6 Ethical Approval and Informed Consent

Ethical approval was not required for this systematic review because it used previously published, de‐identified data. Informed consent was not applicable.

### 2.7 Data Synthesis and Statistical Analysis

Because the included studies consisted of case reports and case series with marked heterogeneity and incomplete reporting, only descriptive analyses were performed. Categorical variables were summarized as counts and percentages and are reported as *n*/*N* (%), where n denotes the number of patients for whom that variable was available. Continuous variables were reported descriptively using ranges. No formal hypothesis testing or meta‐analysis was undertaken due to small sample size, variable diagnostic certainty, and inconsistent reporting across studies. Data extraction, organization, and tabulation were performed using Microsoft Excel. No inferential statistical tests were performed. Therefore, no predefined level of statistical significance was specified.

## 3 Results

### 3.1 Searching Published Literature

**Figure 1:**
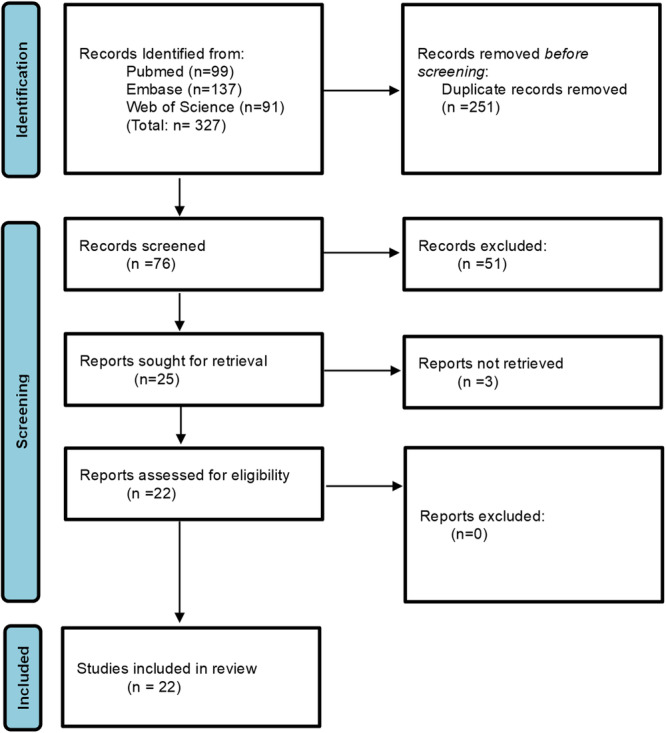
PRISMA flowchart for clinical and genetic spectrum of filippi syndrome.

In our systematic review, a total of 327 articles were identified after a comprehensive literature search. After automatic and manual de‐duplication, 76 citations remained for title‐and‐abstract screening. After that 51 records were excluded at this stage, leaving 25 full texts for retrieval. Three articles could not be obtained despite interlibrary requests, so 22 full‐text reports were assessed for eligibility. All 22 satisfied the prespecified inclusion criteria and were incorporated into the qualitative synthesis. The PRISMA flowchart illustrates the study selection process (Figure 1).

### 3.2 Study Characteristics

This systematic review comprises of 22 studies, with 12 case reports, 8 case series, and 2 editorials reporting cases. The research consisted of a total of 43 individuals who had been diagnosed with Filippi syndrome, including 28 males (65.11%) and 15 females (34.88%). The age at which the patients were diagnosed ranges as early at birth to late at an age of 47 years. The study characteristics of the included studies in our systematic review are shown in Supporting Material 3.

### 3.3 Clinical Presentation

The prevalence of clinical features among reported Filippi syndrome patients, categorized according to Human Phenotype Ontology (HPO) terminology, is summarized in Table 1, highlighting growth abnormalities, craniofacial dysmorphism, limb anomalies, and neurological deficits.

### 3.4 Genetic Changes

Studies that conducted genetic analysis most frequently found mutations in the *CKAP2L* gene, including homozygous and compound heterozygous frameshift, missense, and splice site mutations across several exons. Others included multiple gene deletion at 2q24.3q31.1 encompassing multiple genes [10], heterozygous nine gene gain at 14q11.2 i [11], and *HOXD13 heterozygous* missense mutation in the [12]. Normal karyotypes and/or negative targeted genetic analyses were also reported. Detailed findings of genetic changes can be seen in (Table 2).

**Table 1:** Filippi syndrome features prevalence as per HPO (human phenotype ontology) terminology.

Term	*N*
Growth	
Stunting	35
Microcephaly	34
PNGR	33
IUGR	21
Decrease body weight	24
Craniofacial	
Broad prominent nasal bridge	23
Underdeveloped nasal alae	13
Low‐hanging Columella	2
Broad forehead	6
Prominent forehead	6
Narrow forehead	1
Long philtrum	2
Short philtrum	2
Thin Vermillon border	8
Small primary teeth	9
Micrognathia	4
Eyes	
Telecanthus	13
Small palpebral fissures	10
Upward slanting	4
Downslanting	3
Epicanthic folds	5
Mild ptosis	3
Hypotelorism	2
Strabismus	2
Long lashes	3
Broad thick eyebrows	2
Cardiovascular	
ASD	1
VSD	1
Genitourinary	
Cryptorchidism	11
Renal hypoplasia	1
Renal aplasia	1
Limbs	
Cutaneous Syndactyly toes, fingers Fingers Complete Toe Fingers	34, 32 32
Complete Osseous syndactyly	3
Bilateral clinodactyly	17
Brachydactyly	8
Camptodactyly	2
Aphalangia	2
Bilateral broad great toes	2
Skin, hair, and nails	
High anterior hairline	8
Nervous system	
Global developmental delay	30
Intellectual disability	30
Extremity hypertonia	1
Impaired speech	30
Autism	1
Limb spasticity	
Conductive hearing loss	1
Sensorineural and conductive	1
Seizures	8

**Table 2:** Genetic variants and mutation profiles across Filippi syndrome cases.

Author	Year of publication	Number of patients	Genetic analysis	Mutated gene	Genes product	Types of mutation	Mutated exon
Battaglia [21]	2008	1	Normal male karyotype: 46XY FISH and 7‐DHC analysis gave normal results Array‐CGH at 75 kb was normal Molecular analysis of the coding exon of GJA1 gene showed no sequence variations	NR despite FISH and array‐CGH	NR	NR	NR
Bas [7]	2024	3	1. Next‐generation sequencing (NGS) of the *CKAP2L* gene revealed a homozygous frameshift variant, NM_152515.5: c.554_555del; p. (Lys185ArgfsTer11) 2. Chromosomal microarray analysis returned normal results, but whole exome sequencing identified a homozygous variant in CKAP2L, NM_152515.5: c.1463_1467del; p.(Thr488LysfsTer16) 3. Homozygous novel variant in the *CKAP2L* gene: NM_152515.5: c.981_982del; p. Arg327Serfs*16	Cytoskeleton associated protein 2‐like (CKAP2L), transcript variant 1	Cytoskeleton‐associated protein 2‐like protein	Frameshift mutation	1. Exon 4 2. Exon 9 3. Exon 7
Williams [1]	1999	3	1. Karyotype = 46 XY 2. Karyotype = 46 XX	Only a karyotype was performed	NR	NR	NR
Heron [20]	1995	1	Blood karyotype(G‐banding) = normal Appears to be an independent and possibly homogeneous genetic condition	Only a karyotype was performed	NR	NR	NR
Goyal [4]	2015	1	NR	NR	NR	NR	NR
Patrick [11]	2022	2	Both patient were compound heterozygotes for *CKAP2L* gene paternally inherited variant, c.1169_1173del;p.(Ile390LysfsTer4), and a maternally inherited missense variant, c.2066G > A;p.(Arg689His)	*CKAP2L* gene	Cytoskeleton‐associated protein 2‐like protein	Paternal frameshift deletion, maternal missense mutation	Paternal exon 5, maternal exon 7
Sabir [16]	2019	1	Homozygous for *CKAP2L* gene mutation homozygous variation (c.970dupA p.Thr324Asnfs*2 (exon 4)) in the *CKAP2L* gene,	*CKAP2L* gene	Cytoskeleton‐associated protein 2‐like protein	Frameshift mutation	Exon 4
Lazier [15]	2014	1	Deletion of 2q24.3q31.1(167,170,485–177,613,591) (NCBI36/Hg18).	Multiple genes SCN family genes, DLX1/2, HOXD cluster	Voltage‐gated sodium channels, limbs, and craniofacial development	Deletion	Multiple genes
Yang [22]	2022	1	*CKAP2L* in the proband: c.1759‐2 A > G(NM_152515.5)	*CKAP2L* gene	Cytoskeleton‐associated protein 2‐like protein	Splice acceptor site substitution mutation	Exon 7
Karakaya [6]	2021	1	Homozygous for *CKAP2L* gene, (c.552_555delCAAA, p.Asn184Lysfs*8)	*CKAP2L* gene	Cytoskeleton‐associated protein 2‐like protein	Frameshift mutation	Exon 4
Toriello [5]	1995	1	NR	NR	NR	NR	NR
Sharif [18]	2004	2	Both with a normal karyotype	NR	NR	NR	NR
Capecchi [17]	2018	1	Sanger sequencing reveals Homozygous for *CKAP2L* gene	*CKAP2L* gene	Cytoskeleton‐associatedprotein‐2‐like or Radmis	Frameshift mutation (571dupA (p. Ile191Asnfs*6)	NR
Cabala [14]	2013	1	Karyotyping: Normal male (46, XY)	None	None	None	None
Hussain [2]	2014	11	Sanger sequencing of CKAP2L	*CKAP2L* mutations: 1. c.[571dupA]; [571dupA] 2. c.[571dupA]; [571dupA] 3. None 4. None 5. None 6. c.[2 T > C]; [2 T > C] 7. None 8. c.[554_555delAA]; [554_555delAA] 9. c.[157_485del]; [157_485del] 10. c.[78_79insTT]; [751delA] 11. not analyzed	Cytoskeleton‐associated protein‐2‐like (Radmis)	NR	1. Exon 4 2. Exon 4 6. Exon 1 8. Exon 4 9. Exon 4 10. Exon 1 11. Exon 1
Sandhu [13]	2013	1	NR	NR	NR	NR	NR
Walpole [23]	1999	3	Normal male karyotype; no molecular testing	NR	NR	NR	NR
Orrico [24]	1997	1	Karyotyping: normal 46, XY	NR	NR	NR	NR
de Vries [25]	2016	1	Karyotyping: normal male 46, XY	CREB‐binding protein.	CREB‐binding protein (a histone acetyltransferase)	NR	Exon 30
Fryer [10]	1996	2	NR	NR	NR	NR	NR
Sousa [16]	2013	3	Genome‐wide array comparative genomic hybridization (aCGH): heterozygous gain at band 14q11.2, which included nine genes (REM2, MIR4707, MMP14, PRMT5, C14orf93, LRP10, HAUS4, RBM23, and JUB). in all sisters, mother, and even in brother	Nine genes (REM2, MIR4707, MMP14, PRMT5, C14orf93, LRP10, HAUS4, RBM23, and JUB)	NR	NR	NR
Ravel [18]	2011	1	Karyotyping: Normal 46, XY, FISH analysis showed no deletion of the CREBBP gene on chromosome 16p13.3, Array‐CGH and MYCN were negative.	HOXD13 gene	Heterozygous state p.A6V (c.17 C > T	Missense mutation	NR

*NR, not reported.

### 3.5 Growth

Neonates with Filippi syndrome usually present with microcephaly and are underweight and growth‐restricted at birth. Only a few cases report improvement in centiles in range of third–tenth or greater, while most of them had postnatal growth retardation as well progressing to adulthood. Two overweight patients were reported; interestingly, both had normal height and occipitofrontal circumference [11].

Findings noted on radiology include delayed bone age [17, 21], osteopenia [22], and partial sacral agenesis [12].

### 3.6 Head and Neck

Patients with Filippi syndrome commonly exhibit a broad and prominent nasal bridge, often accompanied by hypoplastic alae nasi. A small depressed nose was reported in only two cases [4, 25]. Other reported nasal features include low‐hanging columella [7, 18], rounded nasal tip [6], pointed nasal tip with slight deviated nasal septum [1], and mild flaring of alae nasi [25]. They may have a prominent forehead (or metopic suture) that can be broad [15] or narrow [16]. Ear abnormalities were reported in only three cases [1, 18, 21].

Primary teeth tend to be small, inconsistently associated with abnormal shaping, and wide interdental space. Incisors are frequently serrated, possibly lacking bimaxillary. Canines are unilaterally short or absent. Overall, there is delayed secondary dentition and poor oral health with carious teeth. Rarely, there are talon cusps [24], congenital absence of all third molars, widespread horizontal bone loss, and an enlarged pulp center [21].

Although lips are usually normal, variations such as a short philtrum accompanied by thin vermillons, a long philtrum [4, 13, 18], short mouth, trismus [1], and asymmetric lips with one higher mouth angle have been reported [18, 23].

### 3.7 Eye

Filippi syndrome patients have relatively telecanthic eyes due to a broad nasal bridge, and rarely hypotelorism. Palpebral fissures are small with or without upward or downward slantation and epicanthic folds. Eyebrows may be broad, thick, and fused (synophrys) with long lashes. Rarely observed findings include strabismus, mild ptosis myopia, and amblyopia [17], deeply set eyes [10], microphthalmia [15, 24], enophthalmos [12], and retinal coloboma [24].

### 3.8 Cardiovascular

Cardiovascular findings were only occasionally seen, including septum secundum atrial septal defect (ASD) [6, 18], ventricular septal defect (VSD) [7, 20], and grade II/VI crescendo‐decrescendo murmur [14].

### 3.9 Genitourinary

Cryptorchidism was the most prevalent genitourinary finding. Infrequently reported were retractile testis [25], micropenis [10], ambiguous genitalia with tiny phallus, hypospadias, flat scrotum [18], and kidney hypoplasia or agenesis, and inguinal hernia [7].

### 3.10 Limbs

The majority of patients exhibited complete cutaneous syndactyly of toes and fingers, mostly of 3–4 digits. Less commonly osseous syndactyly was present. Bilateral clinodactyly primarily involved fifth finger, only one case involving second finger [1].

Rarely reported features include camptodactyly, overlapped fouth toe, radial deviation of index finger at PIP second and third digit splaying [10], bilateral broad great toes, Proportionate brachydactyly [1, 16, 18], talipes equinavarus [7], and indistinct or single palmar creases. Interdigital webbing [12] and nail fusion along with cutaneous syndactyly [18] were unique reported features expanding the phenotype.

Joint issues such as hip, elbow, radial subluxation [13, 14, 24], carpal and tarsal fusions or dysplasias [14], fused metatarsals [18, 23], and exostosis [25] were noted on radiology.

### 3.11 Skin and Hair

Most patients had high anterior hairline along with frontal upsweep of hair and frontal hair growing in different directions. Although mostly normal in color, hair of different shades in the same person and hypopigmented patches were reported [5, 25].

### 3.12 Nervous System

The most frequent neurological findings were mild to severe mental retardation and developmental delay and speech impairment. The majority did not have dystonia, some presented with mild extremity hypertonia, mild truncal ataxia and hypotonia [10], lower limb spasticity [20], spastic cerebral palsy [22], hyper‐reflexia, and extensor plantars [22]. EEG revealed bilateral spike/wave complexes, poor electrical structure, and delayed background activity in individuals who reported having seizures.

Uncommonly, individuals may exhibit moderate autistic behavior [23], combined hearing and speech defect [4, 18, 24], simultaneous conductive and sensorineural hearing loss [24], or isolated conductive hearing loss alone [12]. Magnetic resonance imaging performed in some cases revealed enlargement of subarachnoid spaces and lateral ventricles, megacisterna magna [14, 16, 21], cerebellar vermis hypoplasia [20], and thin posterior corpus callosum [12].

Furthermore, the features extended to include severe atopy and elevated IgE levels in one case [17], two cases; a female and male, both reported poorly‐demarcated hypermelanic macule on the chest, the former one also had talipes equinovarus and behavioral issues [7], and lastly one case reported overlapping features with Rubinstein–Taybi syndrome, Tsukahara syndrome, Tonoki syndrome, Feingold syndrome [12].

### 3.13 Management

Currently, there is no established treatment protocols available with regards to the Filippi syndrome. Management described in the included studies primarily focuses on addressing associated conditions like taking antiepileptic medication to manage seizures [13, 15], syndactyly surgery to release fingers [4], replacing missing teeth [21], gonadotropin‐releasing hormone (GnRH) agonist injections to suppress early puberty [16], alongside supportive therapies such as occupational, physical, and speech therapy to improve motor skills and communication.

### 3.14 Quality Assessment

To carry out a comprehensive analysis of the studies' level of quality, the critical appraisal checklist for case reports and case series developed by the JBI was utilized [26]. Overall, reporting was most complete for demographic description, clinical presentation, and diagnostic assessment, whereas treatment details, postintervention outcomes, adverse events, and follow‐up were frequently missing or unclear. The quality evaluation table for the case reports and case series that we included may be seen in (Tables 3 and 4), respectively. Editorials are not primary research articles and thus were not formally assessed for risk of bias using the JBI checklist.

**Table 3:** Quality assessment of the included case reports using the Joanna Briggs Institute (JBI) critical appraisal checklist for case reports.

Reference (author, year)	A1	A2	A3	A4	A5	A6	A7	A8	Total yes (max. 8)
Battaglia et al., 2008 [21]	Yes	Yes	Yes	Yes	Yes	Yes	No	Yes	7
Heron et al., 1995 [20]	Yes	Yes	Yes	Yes	No	No	No	Yes	5
Goyal et al., 2015 [4]	Yes	Yes	Yes	No	Yes	No	Unclear	Yes	5
Sabir et al., 2019 [12]	Yes	Yes	Yes	Yes	Yes	Yes	No	Yes	7
Lazier et al., 2014 [15]	Yes	Yes	Yes	Yes	No	Not applicable	Yes	Yes	6
Karakaya et al., 2021 [6]	Yes	Yes	Yes	Yes	No	Not applicable	No	Yes	5
Toriello et al., 1995 [5]	Yes	Yes	Yes	No	No	Not applicable	Yes	Yes	5
Cabala et al., 2013 [14]	Yes	Yes	Yes	Yes	No	No	Yes	Yes	6
Sandhu et al., 2013 [13]	Yes	Yes	Yes	No	Yes	No	Yes	Yes	6
Orrico et al., 1997 [24]	Yes	Yes	Yes	Yes	No	No	No	Yes	5
de Vries et al., 2016 [25]	Yes	Yes	Yes	Yes	No	No	Yes	Yes	6
Ravel et al., 2011 [18]	Yes	Yes	Yes	Yes	No	Not applicable	Yes	Yes	6

*Note:* Answer options included: Yes, No, Unclear, and Not applicable; A1: Were patient's demographic characteristics clearly described?; A2: Was the patient's history clearly described and presented as a timeline?; A3: Was the current clinical condition of the patient on presentation clearly described?; A4: Were diagnostic tests or assessment methods and the results clearly described?; A5: Was the intervention(s) or treatment procedure(s) clearly described?; A6: Was the postintervention clinical condition clearly described?; A7: Were adverse events (harms) or unanticipated events identified and described?; A8: Does the case report provide takeaway lessons?

**Table 4:** Quality assessment of the included case series using the Joanna Briggs Institute (JBI) critical appraisal checklist for case series.

Reference (author, year)	A1	A2	A3	A4	A5	A6	A7	A8	A9	Total yes (max. 9)
Bas 2024 [7]	Yes	Yes	Yes	Unclear	Yes	Yes	Yes	No	No	6
Williams et al., 1999 [1]	Yes	Yes	Yes	Unclear	Yes	Yes	Yes	No	No	6
Patrick et al., 2022 [11]	Yes	Yes	Yes	Unclear	Yes	Yes	Yes	Yes	No	7
Sharif et al., 2004 [19]	Yes	Yes	Yes	Unclear	Unclear	Yes	Yes	No	No	5
Hussain et al., 2014 [2]	Yes	Yes	Yes	Unclear	Unclear	Yes	Yes	Unclear	Yes	6
Walpole et al.,1999 [23]	Yes	Yes	Yes	Unclear	Unclear	Yes	Yes	No	No	5
Fryer et al., 1996 [10]	Yes	Yes	Yes	Unclear	Unclear	Yes	Yes	No	No	5
Sousa et al., 2013 [16]	Yes	Yes	Yes	Unclear	Unclear	Yes	Yes	No	No	5

*Note:* Answer options included: Yes, No, Unclear, and Not applicable; A1: Were there clear criteria for inclusion in the case series?; A2: Were there clear criteria for inclusion in the case series?; A3: Were valid methods used for identification of the condition for all participants included in the case series?; A4: Did the case series have consecutive inclusion of participants?; A5: Did the case series have complete inclusion of participants?; A6: Was there clear reporting of the demographics of the participants in the study?; A7: Was there clear reporting of clinical information of the participants?; A8: Were the outcomes or follow‐up results of cases clearly reported?; A9: Was there clear reporting of the presenting site(s)/clinic(s) demographic information?

## 4 Discussion

Filippi syndrome is a very rare autosomal recessive genetic disorder that causes syndactyly, microcephaly, growth retardation, and intellectual disability. It is also associated with abnormal facial features, an enlarged forehead, and a prominent nasal bridge. Filippi syndrome remains exceptionally rare, with only a limited number of published cases reported worldwide [4]. Several case reports and series have been published on this rare syndrome, providing insight into its varied presentations. They also help us understand the possible causes, mechanisms, and patterns behind the clinical findings.

This systematic review comprises 22 studies and provides a comprehensive synthesis of case series and published case reports on Filippi syndrome. The analysis indicates 43 patients with diagnostic ages spanning from birth to 47 years. Abnormal facies, syndactyly, microcephaly, and short stature were among the most consistent clinical findings in reported cases, establishing them as diagnostic characteristics of the disease. Other contrasting anomalies, such as broad or narrow nasal bridges, wide and protruding foreheads, telecanthus, short or long philtrum, palpebral fissures (with or without slanting), irregular ear helix, and varying hair shades or hairline patterns, contribute to the overall appearance of dysmorphic faces and may suggest Filippi Syndrome. Cutaneous syndactyly of the toes and fingers is a predominant feature, most commonly affecting the third or forth digits. Other rare digit abnormalities are also seen in different cases, like osseous syndactyly [2], clinodactyly, camptodactyly, brachydactyly, interdigital webbing, a feature not found in any other patient in the existing literature [12], hypoplastic changes in fingers or toes, and aphalangia [20], which emphasizes the significance of careful limb inspection. Dental anomalies such as small primary teeth, serrated incisors, and the absence of canines or molars further highlight the distinctive craniofacial profile observed in the disease.

A significant insight into the genetic makeup of patients with Filippi syndrome is that *CKAP2L* is the most frequently mutated gene, supported by several articles.

*CKAP2L* (cytoskeleton‐associated protein 2‐like) gene, located on chromosome 2q14.1, is responsible for encoding a protein that plays an important role in mitotic spindle formation and centrosome stability during cell division. It peaks during the G2/M phase of mitosis. Furthermore, this gene is also important for rapidly proliferating stem cells during development, especially of the brain and limbs. Disruption of *CKAP2L* may likely affect centrosome integrity and spindle formation, leading to abnormal cell proliferation [2].

The pathogenesis is mainly due to loss‐of‐function variants in CKAP2L. These mutations result in the absence of proteins at spindle poles and an increase in unorganized spindle poles, leading to defective mitotic spindles. This may present as skeletal defects, such as distinct, coarse facial features, syndactyly, and growth retardation. In addition, loss of *CKAP2L* interferes with normal proliferation of neural stem cells, which appears to be the cause of neurodevelopmental defects, namely microcephaly, intellectual disability, and delayed growth.

According to previously published data, most *CKAP2L* variants are truncating variants, including frameshift, nonsense, and splice‐like mutations. These mutations alter the protein structure by premature termination, leading to a nonfunctional *CKAP2L* protein. Thus, most variants follow a loss‐of‐function mechanism, which is why Filippi Syndrome is described as “centrosomopathy”. Another variant with a missense mutation has also been reported, whose pathogenesis remains less established and needs further research [15].

While current literature suggests loss‐of‐function *CKAP2L* mutations are associated with the pathognomonic phenotype of Filippi syndrome, including syndactyly, microcephaly, growth retardation, and intellectual disability, there is no clear or consistent relationship between specific variants and clinical severity.

### 4.1 Genotype–Phenotype Correlation

A comparison of reported cases in this review shows that molecularly confirmed *CKAP2L* mutations consistently present with microcephaly, syndactyly, and developmental delay. On the other hand, clinically diagnosed cases show much greater variability in phenotype, which may suggest that diagnostic criteria used before distinguishing *CKAP2L* as the causative gene. Most mutation‐positive cases include truncating variants like frameshift mutations, which are associated with a classical clinical presentation. Additional features like seizures, skeletal abnormalities, and endocrine anomalies were also observed among these cases. Several studies have also reported normal genome test results, which may indicate undiscovered disease‐related genes, limitations in testing methodologies, or concerns about interpatient variability. Overall, current published cases do not support a definitive genotype‐phenotype correlation in Filippi syndrome, and further analysis is needed to better evaluate the association.

The skeletal and neurological manifestations are supported by imaging findings, particularly those related to bone deformities, delayed ossification, and cranial structural anomalies. Additional orthopedic complications like hip subluxations, elbow dislocations, and carpal or tarsal fusions accentuate the intricacy of the syndrome. Brain imaging also reveals enlarged brain ventricles and spaces, cerebellar hypoplasia, and thinning of the corpus callosum, which are likely to contribute to the neurocognitive symptoms observed in the patients.

The most persistent neurological symptoms are intellectual disability, developmental delay, and speech impairment, with some also reporting both conductive and sensorineural hearing loss and autistic behavior. The presence of motor delays, spasticity, and hyperreflexia in certain cases suggests a spectrum of motor involvement potentially overlapping with cerebral palsy traits. The EEG abnormalities and reports of seizure‐like activity in several cases further highlight the neurological burden of the disorder, which demands regular EEG monitoring in affected individuals.

Filippi syndrome appears to have a broader systemic impact than previously thought. Cardiac anomalies (e.g., ASDs, VSDs, murmurs) [1, 6], renal agenesis, and genital abnormalities like cryptorchidism highlight the importance of a multi‐systemic workup at diagnosis. The presence of atopy, immune irregularities [17], and pain insensitivity in isolated cases may represent either atypical findings or an underappreciated aspect of the syndrome's profile.

Management remains symptomatic and supportive, with only a few reports detailing interventions such as syndactyly modification, dental prostheses, GnRH medication, or antiepileptic use. This reveals a notable gap in standardized care pathways and calls for a collaboration and multidisciplinary approach, including genetics, orthopedics, neurology, endocrinology, and rehabilitation services, to create better and more structured care for these patients.

This review provides a comprehensive synthesis of the published clinical, radiological, and genetic literature on Filippi syndrome and related overlapping phenotypes. This study consolidates dispersed literature using an integrated approach to understanding the genotype, phenotype, and rare presentations of the disease. The application of PRISMA guidelines to this review ensures its validity and methodological quality.

### 4.2 Limitations

However, several limitations are also reported. First, the condition's rarity limits sample size, and heterogeneity in reporting of case reports prevents easy data extraction. Numerous studies had missing data, such as comprehensive treatment plans, follow‐up results, or genetic verification, and this constrains knowledge of the disease course, its response to therapeutic innovations, and the definitive demonstration of correlations of genotype and phenotype.

## 5 Conclusion

This systematic review provides a comprehensive synthesis of the reported clinical and genetic spectrum of Filippi syndrome and related overlapping phenotypes. The core phenotype is characterized by microcephaly, digital syndactyly, distinctive craniofacial features, growth impairment, and developmental delay, while additional dental, skeletal, neurologic, cardiovascular, and genitourinary abnormalities broaden the recognized spectrum. The results underscore the complexity and variability of the syndrome, necessitating a multidisciplinary approach to diagnosis and treatment. More high‐quality research is needed to improve our understanding and the health of patients.

## Author Contributions

**Muhammad Anas Faheem:** conceptualization, investigation, writing – original draft, writing – review and editing, methodology, formal analysis, project administration, data curation, resources. **Laraib Ghanghro:** conceptualization, investigation, writing – original draft, methodology, writing – review and editing, formal analysis, project administration, data curation, resources. **Ayesha Khan:** investigation, writing – original draft, writing – review and editing, validation, methodology, software, data curation, resources. **Maryam Khalid:** investigation, writing – original draft, methodology, validation, writing – review and editing, software, project administration, data curation. **Salwa Bashir:** investigation, writing – original draft, visualization, methodology, writing – review and editing, formal analysis, software, data curation. **Dania Iqbal:** investigation, writing – original draft, methodology, visualization, writing – review and editing, software, formal analysis, data curation. **Adan Ali:** investigation, writing – original draft, validation, visualization, writing – review and editing, formal analysis, software, resources. **Hussain Haider Shah:** investigation, writing – original draft, writing – review and editing, visualization, methodology, software, resources, data curation. **Sameer Abdul Rauf:** investigation, validation, methodology, writing – review and editing, writing – original draft, project administration, software, resources. **Humaira Kalam:** funding acquisition, writing – review and editing, validation, visualization, methodology, project administration, supervision, data curation.

## Funding

The authors have nothing to report.

## Ethics Statement

Ethical approval was not required for this systematic review because it used previously published, de‐identified data. Informed consent was not applicable.

## Transparency Statement

Corresponding author affirms that this manuscript is an honest, accurate, and transparent account of the study being reported; that no important aspects of the study have been omitted; and that any discrepancies from the study as planned (and, if relevant, registered) have been explained.

## Guarantor Statement

All authors have read and approved the final version of the manuscript. Corresponding author had full access to all of the data in this study and takes complete responsibility for the integrity of the data and the accuracy of the data analysis.

## Supporting information

Supporting File 1

Supporting File 2

## Data Availability

The data that support the findings of this study are available in the Supporting Material of this article. The authors confirm that the data supporting the findings of this study are available within the article and its Supporting Materials.
