# Correction to “Clinical and Genetic Spectrum of Filippi Syndrome: A Systematic Review of Published Case Reports and Case Series”

**DOI:** 10.1002/hsr2.73215

**Published:** 2026-09-14

Faheem MA, Ghanghro L, Khan A, Khalid M, Bashir S, Iqbal D, Ali A, Shah HH, Rauf SA, Kalam H. Clinical and Genetic Spectrum of Filippi Syndrome: A Systematic Review of Published Case Reports and Case Series. Health Sci Rep. 2026 Jul 4;9(7):e72655. doi: 10.1002/hsr2.72655.

The affiliation of the author Adan Ali was incorrectly listed as Affiliation 5 and has been corrected to Affiliation 4.

The online version of this article has been corrected accordingly.

We apologize for this error.

